# *It Takes a Village*: Addressing Community Needs to Implement Firearm Prohibitions for Domestic Violence Protective Order Respondents

**DOI:** 10.1177/08862605251363616

**Published:** 2025-08-27

**Authors:** Kellie R. Lynch, Michael K. Gusmano, Jeff R. Temple, Sarah Lane

**Affiliations:** 1 The University of Texas at San Antonio, USA; 2 Lehigh University, Bethlehem, PA, USA; 3 The University of Texas Health Science Center at Houston, USA

**Keywords:** domestic violence gun laws, community interventions, qualitative research

## Abstract

Prohibiting domestic violence protective order (DVPO) respondents from firearms is an effective strategy to prevent intimate partner homicide. However, DVPO gun laws vary considerably across states, and the implementation of such laws is inconsistent across localities. Local context, such as resource availability, priorities, and politics, differs across types of communities and impacts the implementation of laws. We conducted in-depth key informant interviews with victim service and criminal justice professionals (*N* = 27) working in urban communities, rural communities, and statewide organizations to better understand: (a) what communities need to effectively prohibit DVPO respondents from firearms (i.e., the DVPO gun law) and (b) strategies to help communities view domestic gun violence as a safety issue versus a second amendment issue. We used conventional content analysis to analyze the content of all interviews. Our results revealed a diverse range of community needs for implementing the DVPO gun law; some of which included tangible resources related to personnel, funding, and storage, and other more intangible resources related to community leadership and culture. We also observed competing ideas around strict state leadership directives for how to implement the DVPO gun law versus maintaining community-level autonomy. Finally, many participants stressed the importance of using more effective language to frame the issue of DVPO gun laws as central to addressing domestic violence more broadly, rather than gun control. Regardless of the political landscape and state legislation, communities of all types should be able to make concerted local efforts to limit abusers’ access to firearms, given the danger posed to victims and the broader community. Effective efforts require transdisciplinary collaboration, flexibility, ingenuity, leadership, and funding, thus benefiting from a combined criminal justice and public health approach.

I think it’s really a matter of political will . . . all of the reasons why we shouldn’t try this are unique only to this type of crime. We have never heard that there wasn’t enough room or enough money to store the weapons that are seized from drug dealers. We have never heard that it’s too dangerous to go knock on a drug dealer’s door to get their weapons back. We have never heard that the drug dealers are going to come back and complain about their Second Amendment rights being violated. But those are the things that we constantly hear about this [issue] and I think it really is just an undervaluation of the lives of the people who are most affected by this type of gun violence; primarily, women of color.

-Participant

Having direct access to a firearm is the strongest predictor that a man will kill his female partner (Spencer & Stith, 2020) and the presence of a firearm in an abusive relationship is linked to a five-fold increase in the risk of femicide (Campbell et al., 2003). One potential legal mechanism to prevent this form of deadly intimate partner violence (IPV) includes domestic violence protective orders (DVPOs). DVPOs are civil orders that aim to provide rapid protection to victims and generally involve prohibiting or limiting an abuser’s contact with their victim. While not foolproof, DVPOs are a critical safety tool for victims of IPV and are generally considered an effective way of reducing violence (Logan & Walker, 2009; Russell, 2012; Russell et al., 2025), including femicide (Díez et al., 2017; Vigdor & Mercy, 2006; Zeoli & Webster, 2010; Zeoli et al., 2018). Further, IPV victims who seek DVPOs are typically in severely violent relationships (Logan & Walker, 2009), including experiencing more firearm-related abuse (Lynch et al., 2022).

Under federal law, DVPO respondents are prohibited from possessing firearms and ammunition for the duration of the order (18 U.S.C. § 921 et seq). States vary considerably in the content and enforcement of DVPO gun laws (Cloud et al., 2023; Oliphant & Zeoli, 2024). While some states mirror the federal DVPO regulations, others go beyond the federal gun law and include stronger legislation (e.g., allow weapon seizure on the scene of a domestic violence incident). Still, other states do not have legislation that prohibits DVPO respondents from accessing firearms, which creates jurisdictional limitations as state agencies cannot enforce and prosecute federal laws. The efficacy of DVPO gun laws has received substantive attention in the literature, with strong evidence that DVPO laws reduce the occurrence of intimate partner homicide at the state and local levels (e.g., Díez et al., 2017; Vigdor & Mercy, 2006; Zeoli & Webster, 2010; Zeoli et al., 2018).

## Implementation of DVPO Gun Laws

In addition to investigating their efficacy, considerable research has focused on the implementation efforts of DVPO laws. This work is essential given the variability in the presence of DVPO gun laws across state legislation, as well as the inconsistency around their implementation and enforcement (Cloud et al., 2023; Oliphant & Zeoli, 2024; Zeoli et al., 2019). For example, an analysis of DVPO hearings in North Carolina found that the respondent’s access to firearms was addressed in less than a quarter of the sampled cases; of those, only 39% of perpetrators were ordered to surrender their firearms (Kafka et al., 2022). Guidelines around firearm relinquishment procedures highlight the limitation of enforcement and the potential for noncompliance (Frattaroli & Teret, 2006; Oliphant & Zeoli, 2024; Zeoli et al., 2019). In fact, the efficacy of state DVPO gun laws appear to be contingent on requiring dispossession of the firearm within the legislation (Díez et al., 2017; Zeoli et al., 2018).

Much of the research investigating local efforts to implement and enforce DVPO gun laws has been qualitative in nature and has adopted a key informant approach (e.g., Blackwatters et al., 2023; Lynch et al., 2024; Frattaroli et al., 2021; Lynch & Logan, 2020; Lynch et al., 2018). These methods allow for complex issues to be studied at a deeper level from the perspective of subject matter experts (Marshall, 1996). For example, Frattaroli et al. (2021) interviewed 16 key informants (e.g., attorneys, judges, law enforcement, advocates) about the implementation strategies of prohibiting DVPO respondents from firearms possession. Conducting interviews across four states with differing political climates and DVPO legislation emphasized the range of strategies in implementing DVPO gun prohibition. While key informants from all regions emphasized the importance of assessing compliance with the prohibition, those in states without access to a sales record database described using social media, information from victims, and recorded jail conversations to flag attempts to acquire firearms.

## Importance of Community Context

Understanding community context (e.g., politics and local leadership) is crucial to effectively implementing and enforcing DVPO gun laws. Key components of local context, including infrastructure and resources, political dynamics, and competing priorities, are also likely to differ across communities. For example, relative to urban areas, police in rural communities often receive fewer resources and must cover a larger geographic region. Further, criminal justice professionals in rural areas may view violence against women as a lower priority than other crimes (Logan et al., 2009). In addition, firearms have an important place in rural culture and identity (Ching & Creed, 1997) and are more commonly used for sport/recreation in rural versus urban communities (Blocher, 2013; Boine et al., 2020). Unsurprisingly, a recent PEW Research Center (2023) poll found that gun ownership was much higher in rural (47%) areas compared to suburban (30%) and urban (20%) areas.

There is limited research on DVPO implementation comparing urban and rural communities. In a prior study, Lynch and colleagues administered a mixed-methods key informant survey with 133 professionals from victim services and criminal justice across both urban and rural communities in Kentucky. Urban professionals viewed intimate partner gun violence and homicide as more significant issues than their rural professional counterparts and rated the DVPO gun prohibition as more effective than rural professionals (Lynch & Logan, 2020). Further, urban professionals reported fewer barriers to enforcing the DVPO prohibition and were more likely to report that firearms were discussed in DVPO hearings compared to rural professionals. Professionals from rural areas noted that individuals in rural communities would be hesitant to limit firearm access secondary to concerns about violating respondents’ Second Amendment rights (Lynch et al., 2018). Indeed, while rhetoric around Second Amendment rights is a barrier to expanding gun laws across the country (Spitzer, 2020), it is a particular challenge in majority-conservative states with strong gun culture. After all, politically conservative ideology is tied to pro-gun beliefs and opposition to laws that restrict access to firearms (Celinska, 2007; Dixon et al. 2020).

## Prohibiting DVPO Respondents from Firearms in Texas

The highly populous state of Texas holds a Republican controlled legislative body^1^ and has both multiple large urban areas and many rural counties spread over its vast geography. It is estimated that about 46% of the population of Texas owns a firearm (World Population Review, 2024). Although Texas has an “F” in the Gifford’s Law Center (n.d.) Scorecard for gun laws, DVPO respondents are prohibited from possessing firearms in Texas. Specifically, DVPO respondents, except active, sworn, full-time, paid peace officers, are prohibited from possessing firearms or ammunition under both criminal (TX Penal Code § 25.07[2023]) and family law (TX Family Code § 85.026[2023]). After a temporary DVPO (i.e., ex parte) has been issued and a respondent has been notified of the final DVPO granted by the judge, respondents are prohibited from firearms for the duration of the order, which lasts up to 2 years and can be extended by a judge. However, there are no guidelines for how to implement key aspects of the DVPO gun law, such as relinquishment procedures and assessing compliance, as Texas legislation only requires that a judge “may order” the surrender of a DVPO respondent’s firearms. Compare this vague enforcement to other states that require relinquishment (i.e., “shall order”). Therefore, there is substantial variability among communities around whether and how they enforce the prohibition.

In a previous study (Lynch et al., 2024), we gathered information about implementation efforts of the DVPO gun law across urban and rural communities in Texas as a comparison to emergency protective order (ERPO) gun law implementation efforts in New Jersey. Specifically, we conducted key informant interviews with stakeholders about gun law implementation efforts in their communities. We found that some communities in Texas have formal firearm surrender programs in place that assist respondents with safely relinquishing their firearms and returning them when the DVPO has ended. Other communities essentially ignore the existence of the law and rely on respondents to understand that they are prohibited from firearms, and then choose to comply with this order. Communities that fall in between these two anchors may make some efforts to enforce the law with limited formal protocols. Despite DVPO gun laws’ potential efficacy in preventing intimate partner homicide (e.g., Díez et al., 2017; Vigdor & Mercy, 2006; Zeoli & Webster, 2010; Zeoli et al., 2018), this state-level variability in implementation and enforcement of DVPO mirrors the broader national inconsistency (Cloud et al., 2023; Oliphant & Zeoli, 2024).

Regardless of the reason for failure to implement and enforce DVPO gun laws (lack of knowledge, ability, or willingness), it is critical to develop local strategies that facilitate efforts to disarm DVPO respondents. Indeed, most efforts to implement gun laws occur at the local level (Vernick & Hepburn, 2003), which naturally lends itself to a public health approach. This approach is advantageous as it emphasizes local, community-sensitive strategies and transdisciplinary collaboration among a diverse range of community stakeholders, rather than exclusively using criminal justice stakeholders. However, criminal justice interventions, such as law and policy that address violence, are an important component of the public health approach (Davis et al., 2023). Further, integrating criminal justice interventions is integral for public health strategies for preventing community gun violence (Braga, 2022).

Attempts to diversify the perspective of community stakeholders are also critical given the potential unintentional negative impact of gun law enforcement. For example, Swanson (2020) points out that while ERPO (i.e., “red flag” gun laws) are intended to reduce potential mass shootings (e.g., Wintemute et al., 2019) and suicides (e.g., Swanson et al., 2024), they may also disproportionally negatively impact Black men as ERPOs are commonly initiated by law enforcement (vs. a victim as in the case of a DVPO; Frattaroli et al. 2020; Pallin et al., 2020). This is particularly problematic given the conflicted relationship between the Black community and the criminal justice system in the United States (Thompson, 2019). Further, a recent analysis in Washington state found that the odds of court-ordered firearm relinquishment were 30% to 50% lower for White DVPO respondents compared to Black DVPO respondents (Kafka et al., 2025). These findings suggest judicial protection for White respondents, relative to their Black counterparts, when there are instances of greater judicial discretion with respect to relinquishment orders (vs. mandatory relinquishment). Therefore, public health approaches to gun violence that involve criminal justice interventions must consider potential racial disparities.

A better understanding and leveraging of diverse community needs to develop local strategies for improving policy implementation is a key component to addressing gun violence (Byrdsong et al., 2016). To account for varying needs, priorities, and culture (Blocher, 2013; Logan et al., 2009; Lynch & Logan, 2020; Lynch et al., 2018), these efforts should capture the perspective of stakeholders who work both in criminal justice and victim service sectors from urban and rural areas. Accordingly, we adopted a key informant methodology to assess community needs around implementing DVPO gun laws and local strategies to re-frame the issue of domestic gun violence around safety versus the Second Amendment in sample of urban and rural Texas communities. Specifically, we addressed the following two research questions:

1. What do communities need to effectively implement and enforce DVPO gun laws?
2. What strategies may help communities view domestic gun violence as a safety issue for victims and families versus a second amendment issue?

## Method

### Recruitment and Sample

This study was part of a larger multi-state investigation of the implementation of DVPO and extreme risk protective order gun laws (Lynch et al., 2024) and utilizes the key informant interviews conducted in Texas. We employed a purposive sampling technique, followed by an adaptation of snowball sampling to recruit key informants with knowledge of IPV/homicide and domestic violence gun laws in Texas. This recruitment approach has been adopted in other key informant investigations around domestic violence gun laws (e.g., Lynch et al., 2024; Frattaroli et al., 2021; Lynch & Logan, 2020; Lynch et al., 2018). Specifically, we recruited participants from (a) two large urban areas, (b) three distinct rural areas, and (c) two major domestic violence/sexual assault statewide agencies in Texas. The urban areas were similar (i.e., size, population, and culture) with the exception that one city had a known established DVPO gun surrender program and the other city did not. We selected the rural areas based on recommendations from the statewide participants, as well as their proximity to the selected urban areas, so similar geographic regions of the state were sampled for urban and rural communities. The three rural areas were also generally comparable in size, population, and culture.

We identified the initial group of participants from the two urban communities through their involvement with local gun surrender programs and fatality/high-risk domestic violence teams. We recruited the initial group of rural participants by contacting the central domestic violence/sexual assault agency serving that area and through recommendations from the statewide key informants. We then engaged in snowball sampling by asking initial participants to suggest other appropriate professionals in their communities to include participants who were uniquely positioned to discuss relevant issues (Sadler et al., 2010). Recruitment concluded when no new professionals were identified with relevant expertise/knowledge in the focus of the study. In total, we recruited 37 individuals (23 urban, 11 rural, and 2 state) and 27 completed interviews. Of the 10 who did not complete the interview, four did not respond within the data collection period, three were not permitted to participate by their agency, and three recommended we speak with someone else at their agency. As shown in Table 1, our final sample of key informant participants (*N* = 27) included both criminal justice (e.g., judges, attorneys, law enforcement, parole/pretrial services) and victim service (e.g., advocates, agency employees, counselors) professionals from urban, rural, and statewide agencies.

**Table 1.** Participant Characteristics for Total Sample (*N* = 27).

Characteristic	*n* (%)
Area	
Urban	17 (63.0%)
Rural/statewide	10 (37.0%)
Professional type^a^	
Criminal justice	14 (51.9%)
Victim services	13 (48.1%)
Gender	
Female	20 (74.1%)
Male	7 (25.9%)
Race	
White	17 (63.0%)
Black	2 (7.4%)
Other race^b^	2 (7.4%)
No race specified^c^	6 (22.2%)
Hispanic/Latinx	8 (30.8%)
Age (years)	Mean = 49.0 (Range: 29–71)
Length of time in current position (years)	Mean = 5.2 (Range: 1–22)
Length of time in field (years)	Mean = 21.0 (Range: 2–38)

a Criminal justice professionals included judges, attorneys, law enforcement, pre-trial services, and probation/parole; victim service professionals included domestic violence shelter and other nonprofit victim service agency leadership, and victim advocates.

b Other race category includes participants who self-identified as either Asian/Pacific Islander or “other” race.

c Some participants provided an ethnic identity (i.e., Hispanic/Latinx) without a racial identity.

### Key Informant Interview

We developed a semi-structured key informant interview from a review of the domestic violence gun law literature and adapted some specific questions from Frattaroli et al. (2021). The interview, which lasted about one hour and the questions asked participants about a range of topics including perceptions of the DVPO gun law efficacy, current community efforts to enforce the DVPO gun law, community challenges, strengths, and needs to enforce the DVPO gun law, as well as how to improve efforts to better protect victims of domestic violence. The current study focuses on responses from a subset of questions from interviews with Texas key informants, which were conducted by the first author between July and December 2022.

To address our first research question, we analyzed responses from questions related to community needs (i.e., *What is needed to adequately implement and enforce the DVPO gun law? How have or how could implementation/enforcement challenges be addressed in the community? How can the DVPO gun law be implemented/enforced more consistently?*). To address our second research question, we analyzed responses from the following interview question: *How can you get the general public to view domestic gun violence as a safety issue for victims and families rather than a gun or second amendment issue?* Note that we asked participants to speak about their own communities when answering the interview questions. The two state-level participants spoke about both statewide and community-specific efforts and were particularly familiar with issues in rural communities as their agency provided support to under-resourced communities. All procedures were approved by the authors’ institutional review boards.

### Qualitative Data Analysis

We conducted, recorded, and transcribed all interviews via Zoom. After deidentifying the transcripts and checking them for accuracy, the first author transferred the transcripts into Lumivero’s NVivo for content analysis. The first and fourth author conducted the qualitative analyses using a conventional content analysis approach (Hsieh & Shannon, 2005) and developed/modified the codes during coding rather than using preconceived set of codes (Kondracki et al., 2002). Specifically, the first author read through the transcripts from start to end and then each of the responses to the interview questions of focus (listed above), line by line, to derive preliminary codes. After establishing this initial group of codes, the first author independently coded the 27 transcripts, modifying the codes as needed and adding new codes when responses did not fit into an existing code. The first author used notes and examples of responses to develop code labels. The fourth author then conducted reliability coding for all interviews and met with the first author to review and discuss coding decisions. Any discrepancies were discussed and resolved to reach an agreement.

The codes were then organized into larger themes relevant to the two overarching research questions. Specifically, we organized codes related to the first research question (i.e., what do communities need to implement/enforce procedures related to the DVPO gun law?) into two larger themes—each containing multiple subthemes: community needs related to (a) Resources and Infrastructure and (b) Leadership and Culture. We organized codes to address the second research question (i.e., what are strategies to help communities view domestic gun violence as a safety issue versus second amendment issue?) into the following three themes: (a) Education on the Danger of Guns in Domestic Violence, (b) Focus on Safety Rather than Guns in Messaging, and (c) Raising Awareness of Domestic Violence in Communities. We provide counts (and percentages) of the number of participants who mentioned each theme for the overall sample and as a function of professional type (i.e., victim service vs. criminal justice) and locality type (i.e., urban vs. rural/statewide) in Table 2. We combined the counts for rural and statewide participants given the sampled statewide professionals were very knowledgeable of key issues facing rural communities and provided support to these communities in their efforts to enforce DVPO gun laws implementation.

**Table 2.** Summary of Themes for all Participants and as a Function of Professional and Locality Type.

Theme	Total (*N* = 27(	Professional Type		Locality Type	
		Victim Services (*n* = 13)	Criminal Justice (*n* = 14)	Urban (*n* = 17)	Rural/State (*n* = 10)
**What do communities need to implement and enforce the DVPO Law?**					
1. Resources and Infrastructure					
(a) Funding	10 (37.04%)	5 (38.46%)	5 (35.71%)	7 (41.18%)	3 (30.00%)
(b) Cross-Agency Collaboration	9 (33.33%)	5 (38.46%)	4 (28.47%)	5 (29.41%)	4 (40.00%)
(c) Key Personnel	8 (29.63%)	2 (15.38%)	6 (42.86%)	6 (35.29%)	2 (20.00%)
(d) Relinquishment and Storage	7 (25.93%)	5 (38.46%)	2 (14.29%)	2 (11.76%)	5 (50.00%)
2. Leadership and Culture					
(a) Stronger State Legislation and Leadership	16 (59.26%)	9 (69.23%)	7 (50.00%)	9 (52.94%)	7 (70.00%)
(b) Community Priority and Accountability	15 (55.56%)	8 (61.54%)	7 (50.00%)	9 (52.94%)	6 (60.00%)
(c) Consideration of Local Politics and Culture	14 (51.85%)	7 (53.85%)	7 (50.00%)	8 (47.06%)	6 (60.00%)
**How to get communities to view domestic gun violence as a safety issue versus second amendment issue?**					
1. Education on the Dangers of Guns in Domestic Violence	12 (44.4%)	5 (38.46%)	7 (50.00%)	8 (47.06%)	4 (40.00%)
2. Focus on Safety Rather than Gun Control in Messaging	11 (40.74%)	6 (46.15%)	5 (35.71%)	8 (47.06%)	3 (30.00%)
3. Raising Awareness of Domestic Violence in Communities	10 (37.03%)	6 (46.15%)	4 (28.57%)	8 (47.06%)	2 (20.00%)

*Note*. DVPO = domestic violence protective order.

## Results

### What Do Communities Need to Effectively Implement and Enforce the DVPO Gun Law?

#### Resources and Infrastructure

We further organized needs related to resources and infrastructure identified by participants into four areas: funding, cross-agency collaborations, key personnel, and relinquishment and storage. Each of these is described further below and presented in descending order of the proportion of participants who mentioned each sub-theme.

##### Funding

Funding was the most frequently mentioned resource by participants, with 37% emphasizing the critical need for funding to support the implementation and enforcement of facilitating the relinquishment of DVPO respondents’ firearms. As noted in Table 2, this theme was mentioned by 38.46% (*n* = 5) of victim service versus 35.71% (*n* = 5) of criminal justice professionals and 41.18% (*n* = 7) of urban versus 30% (*n* = 3) of rural/statewide professionals.

Participants highlighted that funding can be a particularly critical difference in the implementation of a DVPO gun law in urban versus rural communities. “*The only reason that we have our gun surrender program is because we had applied for a grant*,” explained a participant from an urban community with an established program. Notably within this sub-theme, participants cautioned against mandating communities to enforce the DVPO gun law without providing adequate support:

It’s [an] extra burden on agencies and personnel. And I think for this to be successful, it needs to be slowly thought out and worked out. And we need to talk to some of these small agencies that might have a sheriff and two deputies [to] see . . . can they do it? And if they can’t, the state needs to come in and help them.

Similarly, a participant said, “*these mandates are great and they’re fantastic, but it doesn’t come with funding. I don’t think communities can afford to do it*.” Another participant reported that funding is the primary barrier to developing a firearm surrender program in their rural community, “*it would take funding to build that program but the funding is not there*.” When discussing the funding involved to build a firearm surrender program in their community, a participant explained, “*there needs to be money to be able to create a program and sustain it because a lot of the programs are driven by the people that champion the cause and it needs to get out of just whoever’s championing it to make sure that it’s law*.”

##### Cross-Agency Collaboration

One third (*n* = 10) of participants mentioned the need for agency collaboration in their responses. This theme was mentioned by 38.46% (*n* = 5) of victim service versus 28.47% (*n* = 4) and 29.41% (*n* = 5) of urban versus 40% (*n* = 4) of rural/statewide professionals.

Participants typically highlighted the need for collaboration across specific criminal justice agencies when discussing this theme, “*definitely a collaboration between the local judge[s], the sheriff, the police, [and] probably the district and county attorney’s office . . . those are the people you would need to come together to really implement it*.” Similarly, a participant noted the critical role judges play in leading collaboration efforts for other agencies, “*the biggest part of that collaboration would have to come from the courts; the judges . . . saying, hey . . . if you don’t follow this procedure, there are consequences. And then law enforcement really following that and going to go do that work and storing* [the weapons].”

Within this theme, multiple participants also explained that these agencies often work in silos and are unaware of efforts at other agencies. This can result in victims falling through the cracks of the system. One participant attributed this isolation to a lack of trust among law enforcement agencies (e.g., metropolitan vs. county) and between law enforcement and victim service agencies, which can lead to passing around the responsibility to enforce gun laws, “*nobody trusts each other . . . you have to build trust in order to [implement the law]*.” Similarly, another participant underscored the importance of collective responsibility among key community stakeholders to effectively disarm abusers, “*for every solution we’ve ever come up with has been met with a whole bunch of excuses. All of those agencies or offices have a role to play in this protocol . . . but we have never had enough buy-in to get everyone to the table at the same time*.” “*It takes a village—everyone’s got to do their part to make sure that everyone is safe*,” a participant stated when explaining the importance of collaboration and communication across agencies in their community.

##### Key Personnel

The need for key personnel to implement and enforce the DVPO gun law was mentioned by about 30% (*n* = 8) of participants. There was more variability across professional and locality types within this sub-theme in comparison to the first two sub-themes. Specifically, 15.38% (*n* = 2) of victim service versus 42.86% (*n* = 6) of criminal justice professionals, and 35.29% (*n* = 6) of urban versus 20% (*n* = 2) of rural/statewide participants discussed this sub-theme.

It is possible that criminal justice professionals were particularly familiar with which personnel are needed to implement the DVPO gun law, given that law enforcement was commonly highlighted as critical personnel. Specifically, participants emphasized that officers would be the primary individuals tasked with enforcing the law. One participant specifically pointed out the additional paperwork and workload of search warrants, “*it is very labor intensive to drive search warrants on a repetitive basis for this. We’re going to have to be able to say what firearm we think is inside the house and why we think it’s still inside that house even though the person has been ordered to surrender through other mechanisms*.” Another participant emphasized the need for additional law enforcement resources in rural communities, “*especially in the smaller communities that . . . don’t have the process in place for . . . law enforcement needs the support not only store the weapons but to facilitate the transfer*.” However, participants also highlighted the need for victim service professionals in implementing the DVPO gun law, such as discussing the value of developing a rapport with victims. For example, one law enforcement officer explained how they work in conjunction with victim advocates,

Because victim advocates don’t wear a badge, they seem more approachable. [They] might build a better rapport with the victims . . . So, I think they learn a lot more about some of what’s going on . . . They might find out, yeah, he’s got a gun and he keeps it hidden over here . . . we might find out more little tidbits and stuff like that that would really help us.

##### Relinquishment and Storage

About 26% of participants noted relinquishment and storage as resources needed to implement the DVPO gun law. Once again, we observed some variability among participants across professional and locality type; 38.46% (*n* = 5) of victim service versus 14.29% (*n* = 2) of criminal justice professionals, and 11.76% (*n* = 2) of urban versus 50% (*n* = 5) of rural/statewide participants discussed this subtheme.

Unsurprisingly, limited storage is likely to be a larger issue in rural areas with fewer resources than urban communities. In fact, concerns over the relinquishment and safe storage of weapons were the most frequently mentioned resource/infrastructure need among rural professionals. Using third-party vendors or storage facilities was mentioned by several participants as a potential solution to challenges with storing guns with law enforcement—especially in rural communities where storage space at law enforcement offices may be limited. For example, a participant described ways that a rural community might get creative to overcome barriers, such as storage space: “*I think if you don’t have a law enforcement agency that has the time and the money and the resources to be able to be the storage and the safe-keeper of the firearms, then there is usually a gun range or a gun store or some other business that knows how to safely store firearms. They know how to inventory*.” Offering this option may address concerns with firearm prohibitions more broadly in the community explained one participant, “*I think that if you want to have a buyback program . . . I think communities will have these conversations*.” However, it should be noted that relinquishment is also a critical issue in urban communities. A judge from an urban community emphasized the need for providing respondents with multiple, clearly defined options for firearm relinquishment as a critical component to ensure compliance with the DVPO gun prohibition. They explained,

We need communities to think together to provide options to these respondents . . . because they could sell their weapons and show [the] bill of sales. But we don’t have any direction really to offer them even if they wanted to comply . . . They’re going to maybe make arrangements with a friend or family member [and] that’s not what model practice would be. But they don’t have any other direction or support around what to do.

Facilitating the relinquishment and storage of firearms is critical, given the DVPO gun law only temporarily prohibits respondents from owning guns for the duration of the order. When discussing the challenges of temporary storage, participants underscored the need for guidance on documenting all firearms received through the courts or law enforcement and ensuring that firearms are properly returned to respondents. One criminal justice professional explained,

Anytime a court of law or law enforcement accept property or seize the property there’s got to be extensive documentation for all that. And particularly with firearms, it gets even more nuanced because they’re supposed to be tracked. There’s supposed to be a separate form for each and every firearm that is collected that has a serial number and it’s reported back . . . so I think there needs to be some really clear templates and examples created that could be utilized by communities.

Further, some participants expressed concern with potential liabilities that come with securely storing weapons, which may deter agencies from wanting to accept surrendered weapons. For example, a participant from a rural community explained, “*some people within the county were afraid that [temporarily storing surrendered weapons] would create some sort of liability; that you know the guns wouldn’t be stored properly or that they would be damaged and then the county would be on the hook*.”

#### Leadership and Culture

We further organized needs related to leadership and culture identified by participants into three areas: stronger state legislation and leadership, community priority and accountability, and the consideration of local politics and culture. We further describe each of these sub-themes in descending order of the frequency which they were discussed by participants.

##### Stronger State Legislation and Leadership

About 59% of participants highlighted the need for stronger legislation and state leadership to signal the seriousness of this issue and mandate that communities implement the DVPO gun law. This sub-theme was discussed by 69.23% (*n* = 9) of victim service versus 50% (*n* = 7) of criminal justice and 52.94% (*n* = 9) of urban compared to 70% (*n* = 7) of rural/statewide professionals.

Some participants argued that having a stronger statewide mandate would make it more difficult for more reluctant communities to refuse to enforce the law, which may explain why this was a particularly common theme among rural professionals. A participant explained, “*it needs to be statewide, not, and you know, up to individual counties or cities to implement. And everyone needs to practice the policy in the process that goes with that. And [it needs to] happen every single time, just not here and there at the discretion of the person*.” Participants, who were often victim service professionals, expressed frustration around the level of judicial discretion to enforce the DVPO gun law in communities that do not have an established gun surrender program. “*I think law enforcement would be extremely reluctant and I don’t think they would do this on their own*,” explained a participant from a rural community when referring to the need for buy-in from the broader criminal justice system in the community. Critical to addressing this issue, a participant described how legislatively changing the DVPO gun prohibition from a “may” to a “shall” order will limit judicial discretion to not enforce the order.

If it comes down from the state, it’s not a local issue . . . the law gives us a lot of guidance and a lot of judgment for ourselves. But there’s certain aspects where we, the law says you shall. That means you will do it. But you have no choice. You have no leeway. You have no discretion . . . If a gun surrender program was to be implemented statewide, then these other smaller jurisdictions . . . they wouldn’t have a choice—it would have to be done.

It is important to note that several participants cautioned against statewide mandates without specific guidelines that explain how to implement a DVPO gun law across different types of communities. One participant explained that developing their rural community protocol of enforcing the DVPO gun law was extremely challenging in the absence of state guidelines, “*we just had to figure it out, you know. Just piecing together knowledge . . . and I mean it was really a lot of hills to climb and a lot of knowledge to gather . . . having an outline of different kinds of protocols that work and steps on how to implement them would be greatly helpful*.”

##### Community Priority and Accountability

About 56% of participants need accountability across different levels to actively enforce the DVPO gun law. This theme was mentioned by 61.54% (*n* = 8) of victim service versus 50% (*n* = 70) of criminal justice professionals and 52.94% (*n* = 9) of urban versus 60% (*n* = 6) of rural/statewide professionals.

While some participants explicitly mentioned law enforcement responsiveness, most who mentioned this theme pointed to the responsibility of the courts to enforce the law. For example, a participant explained, “*if we had courts on board that were would be willing to hear more of these cases . . . if you’re more vigorous in prosecuting these cases and bringing these people back into court if they violate their [PO] conditions . . . the judges are not holding them accountable like they should*.” Similarly, a judge from an urban community emphasized the importance of judges holding compliance hearings to ensure respondents clearly understand the prohibition and surrendered their weapon(s),

I think it has been very beneficial when the judge makes it clear to the respondents what the guidelines are. You know, for example, that part of the firearms surrendering program, that they are to provide proof that they sold the firearms to a reputable vendor. Not just a family member or that they have a receipt that they have put the firearm in possession of the Sheriff’s Department.

Ultimately, participants overwhelmingly placed judges at the center of effectively implementing and enforcing the DVPO gun prohibition and highlighted the difficulties of enforcing the law without their support. “*If a judge isn’t buying into it [the DVPO gun prohibition], you’re not going to get their cooperation. They’re going to let guns fly in their courtroom and they’re not going to order people to give up their guns*,” summarized a participant from an urban community with an established gun surrender program.

It is also noteworthy that participants highlighted the need for the general public and the legislative body to recognize the importance of safe gun ownership and holding those who violate the law accountable. A participant explained, “*we want people to have guns that are responsible and . . . abide by the laws that that we have. If someone is going to go out and continually break the law, then they should not be allowed to have that [gun]*.” Another participant felt the underlying issue was that many communities do prioritize holding IPV perpetrators accountable in comparison to other crimes, such as drug-related crime. This participant explained, “*all of the reasons why we shouldn’t try this [to remove firearms from prohibited owners] are unique only to this type of crime . . . I think it really is just an undervaluation of the lives of the people who are most affected by this type of gun violence; primarily, women of color*.” Similarly, a participant described the need for cultural changes around domestic violence within their community, “*I think that the victim works three times as hard as the perpetrator when police and the judicial system gets involved. I think that needs to change. The onus of domestic violence, I think, is still on the victim . . . I think a huge cultural shift would have to happen*.”

##### Consideration of Local Politics and Culture

About 52% of participants discussed the importance of considering local politics and culture when developing protocols to implement and enforce DVPO gun laws, which is consistent with calls for community-based control over implementation. Specifically, 53.85% (*n* = 7) of victim service versus 50% (*n* = 7) of criminal justice professionals and 60% (*n* = 6) of rural/statewide professionals versus 47.06% (*n* = 8) of urban professionals discussed the importance of considering local uniqueness—particularly in rural communities.

Specifically, participants discussed the different political climates in urban versus rural communities, and that enforcing gun prohibitions could be political suicide in rural communities. For example, “*[the] county sheriff, if he says, I’m going to enforce this and everyone says we’re not voting for you . . . He doesn’t want to lose his job. So, the instant you know it’s politics, and it makes [it difficult]. I don’t agree with it, but I understand it*.” Another participant described how critical it is to build trust and relationships with key community stakeholders when trying to enforce DVPO gun prohibitions in rural communities resistant to enforcing gun laws more broadly due to the political climate. This participant explained:

The majority of the time you really have to build a rapport with somebody for you to even ask that question, especially when it comes to guns . . . and pray that you know the person that you know in the police station or the police department is there and not on vacation when you call. Because if they are, then you know what? It’s not going to be a very good conversation . . . you have to really build relationships [and] that takes a very, very long time.

The same participant also noted that other times it is near impossible to even begin to have conversations about firearms with some rural counties and after years of trying to build a relationship with one particular community, “*it took a new sheriff to actually come in for us to have that relationship with that county*.” However, an urban participant also described the diverse views across judges on the bench within a single urban community, “*you run the continuum of opinions with the judges and they pretty much reflect the diversity in our community and in the way that that’s looked at*.”

Also when discussing the importance of community context, some participants emphasized that efforts to enforce DVPO gun prohibitions should occur at the local level through community responses instead of issuing a statewide mandate. Participants noted how different communities are with respect to resources, priorities, and systems—all things that must be considered when developing enforcement protocols. For example, participants discussed critical distinctions in criminal justice system operations for rural versus urban areas that can explain judicial discretion beyond political beliefs. One participant explained how judges in rural communities may be less likely to order a PO respondent to surrender his or her firearms compared to a criminal defendant, “*the smaller rural areas district judges that are hearing everything like your criminal and your PO and they’re like well, there’s some like really, really scary stuff going on like in these felonious cases and they’re not thinking so much about the protective order cases in the same way*.”

Given these key community differences, some participants were opposed to uniform DVPO gun law implementation procedures across the state. This is a direct contradiction to a previous theme (2a), which called for more leadership and uniformity at the state legislative level. For example, a participant explained:

Have some have some basic guidelines in place, but every local jurisdiction is responsible for coming up with their own actual policies because they’re going to know what they have, what resources they have available, and how that would actually work . . . I think any statewide mandate would honestly be a nightmare, because all the counties are so different

Other participants went as far as to suggest that standard protocols could be broken down by urban versus rural communities, with a participant suggesting, “*I think you would have to have a model for large urban areas moderate urban areas [and] then rural areas*.” Another participant suggested tailoring DVPO gun protocols to communities based on their resources and needs rather than adopting a one-size-fits-allfit’s all approach:

I think that’s why we need to encourage and invest and promote the local communities, especially very different communities . . . to create these transfer protocols. Because we know that what is created in (redacted urban city) is not going to be workable in a really small rural county . . . What do you need? What are the barriers you’re facing? Because if communities are telling us, it’s the lack of legal framework to help us here or it’s a lack of storage, then I think we’d be in a different place to address this issue, both on a resource and funding level.

### What Strategies May Help Communities View Domestic Gun Violence as a Safety Issue Versus a Second Amendment Issue?

#### Education on the Danger of Guns in Domestic Violence

The most frequently mentioned theme addressing strategies to help communities view domestic gun violence as a safety issue versus a Second Amendment issue was increasing education on the danger of guns in domestic violence contexts. This theme was mentioned by about 44% of all participants and discussed by 38.46% (*n* = 5) of victim service versus 50% (*n* = 7) of criminal justice professionals and 47.06% (*n* = 8) of urban professionals versus 40% (*n* = 4) of rural/statewide professionals.

Given that half of criminal justice professionals noted this concern, participants commented on the depth and quality of current educational efforts in the criminal justice system. Further, participants noted that educational efforts should attempt to truly increase understanding of this complicated issue rather than a box-checking exercise. For example, a participant explained that this is particularly important for law enforcement who may not realize why enforcing the law is so important, “*just giving those people an opportunity to learn about what’s really going on instead of just, you know, oh, there’s a new law and this is what’s happening*.” Some participants also pointed to the importance of educating judges, with some suggesting that many judicial officials are unaware of the danger that firearms pose to domestic violence victims. One participant explained, “*we certainly need to better educate our judges and the same applies to our bar as well . . . there should be some consideration to increase improving those requirements for both judges and attorneys*.”

Beyond education efforts in the criminal justice system, participants emphasized that changing the conversation around domestic violence and firearms within the community can only come with education. “*I think if people understood the risks associated with people who have protective orders issued against them who are found to have weapons, and who have used weapons and the likelihood that they will continue to do that. I think more people would be willing to support prohibition*,” a participant explained. Similarly, another participant responded,

I think a lot of the misunderstanding of family violence is . . . the inability of the victim to leave the perpetrator on a lot of occasions. It’s easy for somebody who’s not in a situation to say, why don’t you just leave? She has kids, she has no shelter, no job, no income, no whatever it may be . . . And if they have that understanding . . . [of] the dynamics of family violence, the more likely [they are] to understand how firearms would come into play in these situations.

#### Focus on Safety Rather than Gun Control in Messaging

About 41% of participants discussed multiple strategies focused on messaging to shift the conversation away from Second Amendment rights and gun control to safety. This theme was discussed by 46.15% (*n* = 6) of victim service professionals versus 35.71% (*n* = 5) of justice system professionals and 47.06% (*n* = 8) of urban professionals versus 30% (*n* = 3) of rural/statewide professionals.

Some participants suggested that framing the issue around holding criminals accountable and emphasizing that law abiding citizens will not be impacted by the DVPO gun law may be effective. One participant stated, “*we’re talking about enforcing the laws that are on the books for people that are not supposed to have guns. We’re not asking to take away your gun*.” Similarly, another participant explained, “*this [domestic violence] is a criminal act and when you are found to have committed a criminal act you lose certain constitutional rights, your freedom. I mean, we can put you in jail for committing a criminal act so why can’t we take your gun away*?” Multiple participants also recommended framing firearm surrender programs to help offenders follow the law. For example, a judge in an urban community explained how they present the issue in their court, “*I think shifting the focus from what the victim can do towards what the offender can do . . . it’s not just, hey, let’s hold him accountable when he does something wrong. But, hey, let’s also provide some resources if the offender wants to make some changes*.”

Another participant suggested that shifting the conversation away from domestic violence onto broader community safety issues, such as mass or school shootings, might be more effective in getting the public to agree with legislation that restricts abusers’ access to guns. For example, “*maybe the tactic is making that connection between domestic violence homicide and mass shootings or other things that seem less palatable to the general public. You know there is a really high degree of tolerance for violence against women or within the family that doesn’t exist for other things*.” Other participants suggested that the intersection between guns, domestic violence, and mental health should be emphasized, given that so many people in the community experience or know someone who has had mental health issues. However, one participant cautioned against centering mental health in issues around gun violence and gun laws, “*there’s clearly an intersection here with mental health, but how do we [not] further stigmatize people with mental health? Because most people with mental [health] problems don’t go and kill people. So yeah, I think it’s a tough line to walk*.” Finally, some participants suggested approaching the issue from a logical standpoint given the severity of gun violence compared to violence involving other weapons. “*This is not a second amendment issue . . . we’re not going after your guns. But if you compare the potential fatality [of] someone coming at you with a knife versus someone coming at you with a gun . . . you have a better chance of surviving*” a participant explained.

#### Raising Awareness of Domestic Violence in Communities

Another theme to emerge centered on raising awareness of domestic violence more broadly within the community, which was discussed by about 37% of participants. This theme was mentioned 46.15% (*n* = 6) of victim service versus 28.57% (*n* = 4) of criminal justice professionals and 47.06% (*n* = 8) of urban versus 20% (*n* = 2) of rural/statewide professionals—perhaps reflecting populations of stakeholders that are currently engaging in more community awareness efforts and advocacy around this issue.

One participant explained that ignoring the issue of domestic violence is a way to avoid addressing the issue within the community, “*I think domestic violence is something that people don’t want to talk about and they think that it’s not going to happen to them or that it doesn’t affect the community. So they feel like it’s something that’s going to go away*.” “*It’s still very taboo*,” stated another participant when discussing how domestic violence is viewed in their community. Multiple participants emphasized the importance of sharing the experiences of victims with the community to increase understanding of the issue. One participant explained,

One of the big issues with domestic violence is that it is kept secret. So, if there is more awareness and understanding of the actual problem of domestic violence [and] if there were campaigns that actually gave women the platform to speak out and . . . for victims to actually share their stories especially when it comes to firearms . . . I think that’s [the secrecy] part of the reason why people don’t understand how connected firearms are to violence.

When discussing specific ways to increase community awareness around domestic violence, participants highlighted the use of local and social media. For example, “*we work very hard with our media consultants on getting this into the media so that it’s being talked about in the press and it’s being accurately captured and reflected sort of what our aims and goals are. And so, I think that there’s a big role that media plays*.” A participant suggested that domestic violence awareness efforts should be prioritized throughout the year and not just during domestic violence awareness month in October. Another participant explained grassroots efforts to overcome resource limitations associated with advertisements, “*public service announcements take money and time. But we could go to different churches . . . just a little ten-minute [talk] just try to educate and be available to answer questions*.” Finally, some participants suggested that domestic violence should be treated as a public health issue to challenge the notion that it is a private matter. For example, “[*we need to] treat it like a public health issue and support raising awareness like we would a public health crisis because that’s what it is . . . until that happens, you know, unfortunately, it’s still going to be viewed by many as an issue that happens within the four walls of your home*.”

## Discussion

We adopted a qualitative key informant approach to better understand what urban and rural communities need to effectively implement DVPO gun laws, as well as strategies to help communities view domestic gun violence as a safety versus Second Amendment issue. Our results revealed a diverse range of community needs for implementing the DVPO gun law, including tangible resources related to personnel, funding, and storage, and intangible resources related to community leadership and culture. We also observed competing ideas around strict state leadership directives for how to implement the DVPO gun law versus maintaining community-level autonomy. Some participants argued that strong state leadership is critical to ensuring that communities are implementing the DVPO gun law, while others argued against a one-size-fits-all approach. Finally, many participants stressed the importance of using more effective language to frame the issue of DVPO gun laws. Rather than focusing on these interventions as gun laws or “gun control,” it would be more effective to emphasize their role in addressing the broader issues of domestic violence and community safety.

We also descriptively examined the occurrence of these themes across participants’ professional (i.e., victim service vs. criminal justice) and locality (i.e., urban vs. rural/statewide) type. We not only found many similarities across these sub-populations of stakeholders but also observed some notable differences. For example, participants in rural communities particularly emphasized concerns around storage of surrendered firearms, cross-agency collaboration, and the need for stronger state leadership on mandating firearm removal. Victim service professionals not only echoed similar priorities but also emphasized the need for raising community awareness of domestic violence more broadly when framing the importance of DVPO gun laws around safety. Urban professionals particularly recognized the value of tangible resources such as funding and key personnel for implementing DVPO gun laws. Similarly, criminal justice professionals particularly highlighted the need for sufficient personnel but also more education for justice system professionals on the dangers of firearms in the context of IPV. While many issues cut across community type and stakeholder sectors, differences in urban versus rural communities reflect the need to consider localized firearm policy due to differences in culture and resources (Blocher, 2013). Differences in the perspectives of victim service versus criminal justice professionals reflect the two “arms” (i.e., carceral vs. therapeutic) of implementing IPV policies (Sweet, 2021). Both are necessary, but are not always complementary. Below, we expand further upon key implications of the current work and include recommendations for improving DVPO gun policies and intimate partner homicide prevention more broadly.

### Key Findings and Implications

#### State Mandates Versus Local Efforts

We observed competing ideas around strict state leadership directives for how to implement the DVPO gun law versus maintaining community-level autonomy for implementing the law. Some participants argued that strong state leadership and legislation are critical to ensuring that communities are implementing and enforcing the DVPO gun law, with some participants explicitly stating that the implementation of such laws “*is not a local issue*.” Despite this view, most efforts to implement gun laws are achieved at the local level (Vernick & Hepburn, 2003) and participants also noted problems with a singular implementation protocol as communities differ drastically across the state. One participant suggested that if the state were to develop best practice protocols, there should be separate guidelines for urban versus rural communities. This idea is similar to Blocher (2013)’s firearm localism, which suggests that firearm-related policy may be more effective if it differs across urban and rural communities given these communities often differ in culture, justice system structure, and resources. The division over the issue of state vs. local control mirrors political and legal debates that have taken place across the country. But in contrast to our findings in this case, advocates for state preemption of local gun laws in the U.S. have more frequently been championed by Republican controlled state legislatures in efforts to preempt city efforts to enact stricter gun control measures (Blocher, 2021; Fowler & Witt, 2019). Pomeranz and colleagues found that “the majority of states used preemption as a tool to support policy frameworks favoring gun rights” (Pomeranz et al., 2021). In our study, advocates for a more uniform implementation of DVPO laws were more likely to call for reduced local autonomy.

While we do not advocate for rural communities to have different firearm policies and underscore the importance of strong state-level firearm legislation, the specifics around the implementation of the DVPO gun law should reflect what works best within each community. It is ideal for a state not only to possess strong legislation that specifically outlines details around repossession to support local enforcement of the law but also to provide communities with enough autonomy to implement the law in a way that makes sense. However, that is often not the case. We do, however, advocate for the need to keep rural communities in the spotlight in investigations of and conversations around gun violence prevention. An analysis of U.S. gun deaths between 2011 and 2020 revealed that rural areas had a 37% higher rate of gun deaths per capita compared to urban cities (Reeping et al., 2023). This was largely driven by higher incidences of suicide involving firearms in rural areas versus urban areas—a phenomenon observed in prior research (Branas et al., 2004). While urban communities may be more likely than rural areas to implement programs to implement the DVPO gun law, it should not be assumed that gun violence and domestic violence are unimportant issues in rural communities.

Further, strategies to increase the enforcement of gun laws should consider the starting point of many rural communities that may be starting with little to no existing infrastructure and strong resistance to enforcing gun laws. Our stakeholders in rural communities advocated the need for blueprints and guidelines for how to start a firearm surrender program or DVPO gun law enforcement procedures from scratch. Sharing success stories from communities similar in size, culture, and resources for how to start a firearm surrender program from the ground up would be valuable. Relatedly, the importance of funding and infrastructure for key resources such as personnel and storage cannot be overstated. Assisting rural communities with securing funding for the infrastructure needed to implement the law may bolster against opposition arguing that an already under-resourced justice system cannot take on additional efforts to enforce DVPO violations and reduce tension stemming from unfunded mandates.

#### Flexibility in Implementation

Critically, communities are at different stages of implementation or readiness to implement DVPO gun law procedures and addressing community needs is a fluid process. What might work in a “model city” may not work elsewhere or in a less resourced area; therefore, endeavors to increase efforts to implement and enforce a DVPO gun law necessitate meeting communities where they are. A salient example of this flexibility in our interviews was having multiple de-possession strategies, such as allowing sales to third parties, rather than relying on relinquishment to law enforcement. De-possession procedures are critical to the efficacy of DVPO gun laws (Díez et al., 2017; Zeoli et al., 2018), making them a top priority for local implementation efforts. A key resource identified to facilitate de-possession is weapon storage, given that DVPO is a temporary gun prohibition. While a traditional storage mechanism involves law enforcement receiving and storing weapons, some communities are unable or unwilling to ask law enforcement to take on additional storage. Participants discussed ways to overcome this barrier by collaborating with firearm retailers or via buy-back programs and presenting evidence of the sale of a firearm. These mechanisms may limit respondents’ loaning their firearms to friends/family and improve compliance with the prohibition. However, the efficacy of gun buy-back programs for targeting high-risk gun owners and reducing future violence in the United States is limited (see Charbonneau, 2023 for a full discussion), and using third-party sales in the context of DVPOs is an area in need of empirical investigation.

While victims play a critical role in documenting and reporting DVPO violations (Logan & Walker, 2009), participants in our study suggested using the enforcement of DVPO gun laws as a mechanism to help offenders achieve compliance. One participant noted that if compliance is a primary goal, then the system should try to set DVPO respondents up for success and provide them with as many options as possible to dispose of their firearms. Increasing comprehension of the legal process, reducing barriers for compliance, and maximizing helpfulness of court actors have been identified as factors that facilitate compliance more broadly in domestic violence cases (Davis, 2019). Our interviews emphasized the critical role of judges and the courts when developing broader implementation strategies to improve compliance versus placing the majority of responsibility on law enforcement. Many participants reported that without judicial buy-in, it would be very difficult to implement and ultimately enforce DVPO gun laws—especially in states whose legislation specifies that a judge “may” order the surrender of DVPO respondents’ firearms versus “shall” order.

#### Framing Domestic Violence as the Center of the Issue

Finally, many participants stressed the importance of bringing domestic violence to the forefront of conversations, as the DVPO gun law is not just a “gun control” issue. Re-framing gun violence as a broader safety issue that requires collaboration is consistent with the public health approach to gun violence (Hemenway & Miller, 2019) and may also help build inter-agency trust and ease tensions around whose responsibility it is to implement laws that restrict abusers’ firearm access. However, some participants argued that IPV is simply not taken as seriously as other forms of violent and drug-related crimes, so it might be advantageous to focus on the link between mass shootings and domestic violence to motivate agencies to enforce DVPO gun laws (Everytown for Gun Safety, 2019; Geller et al., 2021; Gu, 2020). One participant went as far as to suggest that other gun violence, such as school shootings, are less “palatable” to the public than IPV and other forms of domestic violence, which goes back to an underlying issue IPV is not be taken as seriously as it needs to be in many communities (Logan et al., 2009). Further, IPV is further delegitimized among victims of color, as Black women experience additional barriers of discrimination and “legitimacy” as victims (Simmons, 2020) despite experiencing both fatal and non-fatal IPV at disproportionate rates (Petrosky et al, 2017; Smith et al., 2017). As participants pointed out, this reality provides additional complexity for advocating to legislators and communities the importance of taking action on policies that protect IPV victims. While this is a difficult systemic issue to fully address, it underscores the need for community-driven prevention efforts to include a diverse perspective of stakeholders who recognize and value the populations that are significantly impacted by IPV and the unique barriers they face when navigating the criminal justice system. Additionally, it is critical for criminal justice agencies to understand that IPV and other forms of domestic violence (e.g., family violence) make up a significant proportion of violent crime in the justice system (e.g., Hartley et al., 2025) and should not be treated as niche and unimportant issues.

Our participants, particularly victim service professionals, also emphasized the need to keep domestic violence victims at the forefront of the issue, as their safety should be prioritized above political or system rhetoric. One participant noted that victims must work “*three times as hard*” as offenders in the current justice system, which has been echoed in Sweet (2021)’s work on how IPV victims navigate systems and policies during and after their abuse. Specifically, Sweet (2021) notes that “to do well in the legal system, they have to convince powerful authorities that they are legitimate victims (p. 13).” In this vein, it is important to consider that carceral approaches to addressing IPV, which include civil remedies such as DVPOs, have significant limitations for adequately addressing the needs of victims (Poor, 2023). Out of fear of further angering their abuser, the firearm prohibitions that come as a condition of DVPOs may deter some victims who would otherwise find DVPOs helpful (Lynch & Logan, 2018). Similarly, contacting law enforcement can endanger rather than protect victims (Lippy et al., 2020; Wolf et al., 2003) and leave victims feeling unheard and blamed (Mookerjee et al., 2015). These issues are potentially exacerbated among certain populations, such as Black victims, who have a tenuous history of distrust with law enforcement (Monterrosa, 2019).

In sum, while pursuing a criminal justice response may be helpful for some IPV victims, these interventions can also be ineffective and harmful for victims (Derr et al., 2025; Monterrosa, 2019; Sweet, 2021), which emphasizes the need for non-carceral responses to IPV. Despite the potential life-saving benefits of DVPO gun laws for IPV victims (Díez et al., 2017; Vigdor & Mercy, 2006; Zeoli & Webster, 2010; Zeoli et al., 2018), full consideration of the potential unintended negative consequences of gun law enforcement (Swanson, 2020) is critical to effective conversations around preventing intimate partner homicide.

### Limitations and Future Directions

Our findings should be considered in light of several limitations. First, the perspectives of participants in our sample are not representative of the entire state of Texas as we did not employ a random sampling procedure and did not sample from all regions or counties of the state. Though our sample size is consistent with other qualitative key-informant investigations of gun laws (e.g., Frattaroli et al., 2021; Pallin et al., 2021) and in-depth qualitative investigation does not lend itself to providing representative population estimates (Tolley et al., 2016). Further, the themes and strategies from our results may be used to improve DVPO gun law implementation in other states that lack strong domestic violence gun laws. Nevertheless, the generalizability of these findings is limited given the selection bias from this purposive sampling procedure. A cluster sampling procedure in future efforts, for example, would allow for better representation of stakeholder perspectives across a large state like Texas.

Second, although we sampled from multiple urban and rural areas, our sample of professionals was lacking in representation from certain populations. For example, the sample was majority female, and White or Hispanic with limited representation from Black professionals. Therefore, the needs and strategies discussed in the findings may not capture critical needs in the community for Black victims or offenders, who face significant discrimination and inequality in the justice system (Decker et al., 2019; Hulley et al., 2023; Monterrosa, 2019; Thompson, 2019) and disproportionate rates of IPV (Gillum, 2019). Civil order gun laws (e.g., ERPO gun laws) in particular, despite the intention to reduce violence and suicide, may have unintentional negative consequences for Black men (Swanson, 2020). Further, researchers have observed leniency toward White DVPO respondents in comparison to Black DVPO respondents when ordering firearm relinquishment (Kafka et al., 2025). While Zeoli et al. (2018) observed a 13% reduction in intimate partner homicides when states prohibited DVPO respondents from firearms, a subsequent re-analysis found that state-level DVRPO gun laws were associated with a significant reduction in intimate partner homicide for White victims but not Black victims (Wallin et al., 2022). This difference highlights a critical weakness in the efficacy of legal interventions for populations who are at a higher risk for serious injury or death, given that Black women are murdered by an intimate partner at a higher rate than White women (Petrosky et al., 2017). Therefore, there is a need to adopt an intersectional lens when looking at the efficacy of efforts to prevent intimate partner homicide in future efforts (Gray et al., 2024).

Third, while we sampled participants from both urban and rural communities, we did not conduct a formal comparison of community needs as a function of community type. Further, we had a greater representation of professionals from urban areas, which was not only due to the greater number of eligible professionals in urban areas but also a stronger reluctance for rural professionals to participate in interviews about implementing gun laws. In particular, representation from rural criminal justice professionals (e.g., law enforcement and judges) was particularly difficult, but a challenge also observed in other key informant research (Lynch & Logan, 2020; Lynch et al., 2018). Future research should also consider including suburban communities when recruiting key informants, as we did not sample from such areas despite the existence of multiple suburban areas in the state. The classification of communities is more nuanced than binary urban versus rural (Bibby & Shepherd, 2004), and there is substantive variability within different types of rural areas in particular (Rennison & Mondragon, 2022). Relatedly, while we descriptively presented the proportion of urban versus rural/statewide and victim service versus criminal justice professionals who discussed each theme, we caution against overinterpreting statistical meaning in these results, given the sample size and qualitative nature of this study.

Fourth, we focused exclusively on the implementation of the legislation that prohibits DVPO respondents from possessing firearms. However, this is not the only gun law that is relevant to IPV and intimate partner homicide, as both federal law and Texas state law also prohibit domestic violence misdemeanants from possessing firearms. However, there is little research on the implementation of domestic violence misdemeanant gun laws and the established firearm surrender program in one of our sampled urban communities was exclusively for civil DVPOs and did not extend to criminal courts. Implementation efforts for gun laws in the criminal justice system are warranted, given Zeoli et al. (2018)’s state-level analysis found that prohibiting firearms for those convicted of any violent misdemeanors (i.e., not specifically domestic violence) was associated with a 23% reduction in intimate partner homicide.

## Conclusion

While firearm access is a key risk factor for intimate partner homicide (Campbell et al., 2003; Spencer & Stith, 2020), firearms also play a critical role in non-fatal IPV and are often used to intimidate, control, and threaten victims (Adhia et al., 2021; Kafka et al., 2021; Lynch & Logan, 2018; Sorenson, 2017; Sorenson & Schut, 2018; Sorenson & Wiebe, 2004). Even after accounting for non-firearm-related abuse, firearm-related IPV has been linked to post-traumatic stress disorder symptoms (Sullivan & Weiss, 2017) and neurological health issues (e.g., headaches, chronic pain; Lynch & Jackson, 2021). Therefore, measuring the impact of firearm legislation on IPV victims should go beyond metrics of intimate partner homicide. Relatedly, firearms are just one tool to control and kill victims and that efforts to prevent IPV and intimate partner homicide must go beyond enforcing firearm legislation. Nevertheless, enforcing firearm relinquishment of DVPO respondents substantively reduces the occurrence of intimate partner homicide (e.g., Díez et al., 2017; Vigdor & Mercy, 2006; Zeoli & Webster, 2010; Zeoli et al., 2018), making DVPO gun laws a worthwhile legal intervention. Regardless of the political landscape and state legislation, communities of all types should be able to make concerted local efforts to limit abusers’ access to firearms, given the danger posed to victims, their families, and the broader community. Though best practices or ideals may not be an option for many communities, effective local efforts require flexibility, ingenuity, leadership, and funding. Creating a culture that prioritizes the prevention of domestic violence and homicide is also critical to keeping guns out of the hands of abusers, as these policies ultimately center around victim safety, not Second Amendment rights.

## Notes

1.
https://legdir.capitol.texas.gov/about.aspx
